# Lutein Maintains Bone Mass In Vitro and In Vivo Against Disuse-Induced Bone Loss in Hindlimb-Unloaded Mice

**DOI:** 10.3390/nu16244271

**Published:** 2024-12-11

**Authors:** Yuki Tanaka, Tsukasa Tominari, Masaru Takatoya, Daichi Arai, Moe Sugasaki, Ryota Ichimaru, Chisato Miyaura, Chiho Matsumoto, Sihui Ma, Katsuhiko Suzuki, Michiko Hirata, Florian M. W. Grundler, Masaki Inada

**Affiliations:** 1 Cooperative Major of Advanced Health Science, Tokyo University of Agriculture and Technology, 2-24-16 Naka, Koganei 184-8588, Tokyo, Japan; 2 Department of Biotechnology and Life Science, Tokyo University of Agriculture and Technology, 2-24-16 Naka, Koganei 184-8588, Tokyo, Japan; tominari@ncnp.go.jp (T.T.); miyaura@isc.chubu.ac.jp (C.M.); c-matsu@cc.tuat.ac.jp (C.M.); hirata@cc.tuat.ac.jp (M.H.); 3 Faculty of Sport Sciences, Waseda University, 2-579-15 Mikajima, Tokorozawa 359-1192, Saitama, Japan; masihui@toki.waseda.jp (S.M.); katsu.suzu@waseda.jp (K.S.); 4 Institute of Crop Science and Resource Conservation, University of Bonn, Karlrobert-Kreiten-Strasse 13, 53115 Bonn, Germany; grundler@uni-bonn.de; 5 Life Science Inada Team, Institute of Global Innovation Research, Tokyo University of Agriculture and Technology, 2-24-16 Naka, Koganei 184-8588, Tokyo, Japan

**Keywords:** lutein, beta-cryptoxanthin, astaxanthin, osteoclast, bone resorption, bone formation, disuse osteoporosis

## Abstract

Background: Lutein, a carotenoid, exhibits various biological activities such as maintaining the health of the eye, skin, heart, and bone. Recently, we found that lutein has dual roles in suppressing bone resorption and promoting bone formation. In this study, we examined the effects of lutein in a disuse-induced osteoporosis model using hindlimb-unloaded (HLU) mice. Methods: Osteoclast differentiation was assessed by coculturing mouse primary osteoblasts and bone marrow cells or culturing a mouse osteoclast precursor cell line. The bone-resorbing activity was determined by mouse calvarial organ cultures. An in situ docking simulation was conducted to reveal the interaction of lutein and IκB kinase (IKK) β protein. HLU mice were fed a 1% lutein-containing diet for two weeks, and the femoral bone mass was measured by μCT. Results: Osteoclast differentiation is significantly inhibited by lutein, astaxanthin, and β-cryptoxanthin. In contrast, only lutein promoted osteoblastic calcified bone nodule formation. To elucidate the molecular role of lutein, we functionally analyzed the NF-κB complex, a molecule involved in bone metabolism, especially in osteoclasts. Docking simulations showed that lutein binds to IKK, thus inhibiting the activation of NF-κB. In a cell culture analysis, the phosphorylation of p65, the active form of NF-κB in osteoblasts, was suppressed by lutein treatment. In vivo, a μCT analysis of the bone microarchitecture showed that lutein improves several bone parameters while maintaining bone mass. Conclusions: Lutein is effective in maintaining bone mass by controlling both bone resorption and formation, which is applied to prevent disuse-induced osteoporosis.

## 1. Introduction

Bone homeostasis is preserved by an equilibrium between osteoclastic bone resorption and osteoblastic bone formation [1]. An excessive osteoclast function results in various bone diseases, such as periodontal disease, osteoarthritis, and osteoporosis [2]. In these diseases, osteoclast differentiation and function are stimulated by the crucial molecule known as the receptor activator of the NF-κB ligand (RANKL). The interplay between osteoblastic RANKL and RANK in osteoclast precursor cells controls osteoclast differentiation and subsequent bone resorption [3]. Pro-inflammatory mediators such as interleukins (ILs) and prostaglandins (PGs) increase the production of RANKL in osteoblasts, thereby inducing inflammatory bone resorption [4,5,6]. Anti-osteoporotic agents, such as bisphosphonates, selective estrogen receptor modulators, anti-RANKL antibodies, and parathyroid hormones, are effective in restoring bone loss in disuse osteoporosis by targeting osteoclast precursor cells, osteoclasts, and osteoblasts [7]. Physical disabilities like long-term bed rest or cast immobilization can lead to disuse osteoporosis, characterized by a lower bone mass and increased fracture risk due to excess bone resorption. Hindlimb-unloading (HLU) mice, also known as tail-suspension mice, are widely used as experimental models of disuse-induced osteoporosis. However, there are few reports on the application of nutrients in these models.

In a super-aged society, nutritional supplements containing phytochemicals, such as carotenoids and flavonoids, are a current trend in maintaining bone health. Several studies have shown that phytochemicals, such as carotenoids and flavonoids, are effective in improving bone health in mice [8,9,10,11,12]. Our previous studies have demonstrated the beneficial effects of various phytochemicals on bone metabolism in mouse cell cultures and bone disease models. Several flavonoids including polymethoxyflavones and catechins [13,14] and carotenoid β-cryptoxanthin [15] suppressed LPS-induced osteoclast differentiation and periodontal alveolar bone loss in mice. Polymethoxyflavones suppressed estrogen deficiency-induced bone loss in OVX mice [16]. Feeding with isoflavones and the combination of soy isoflavones and resveratrol can prevent bone loss in HLU mice [17]. Other studies have shown that β-cryptoxanthin and lycopene, carotenoids found in citrus fruits and tomatoes, respectively, exhibit the anti-osteoporotic effect [10,18,19,20,21]. In addition, the dietary intake of carotenoids and high serum carotenoids have a positive effect on bone health in postmenopausal women [22,23,24].

Lutein is one of the major carotenoids found in fruits and vegetables and is a beneficial phytochemical for eye health [25]. To maintain eye health, lutein is thought to continuously accumulate in the eye and embed in cell membranes, scavenging reactive oxygen species (ROS) and/or boosting the activities of antioxidative enzymes [26,27]. We found that lutein inhibits bone resorption and promotes bone formation in bone tissues [28]. Additionally, administering a 1% lutein-containing diet for 4 weeks increased the peak bone mass in growing mice [29], while local lutein injections ameliorated lipopolysaccharide-induced periodontal bone resorption in mice [30]. Several reports indicated that ovariectomized (OVX) mice show estrogen deficiency-induced bone loss related to increased oxidative stress and inflammation [31,32,33,34]. Indeed, the lutein intake in OVX rats mitigates bone loss by downregulating the expression of NF-κB and the nuclear factor of activated T cells, cytoplasmic 1 (NFATc1), while upregulating the expression of nuclear factor erythroid 2-related factor 2 (Nrf2) [35]. In human studies, Bovier et al. found a substantial correlation between the lutein and zeaxanthin status and increased bone mass in the femur and lumbar spine in 63 healthy individuals [36]. Regu et al. reported that the dietary intake of lutein and zeaxanthin is positively associated with the total hip bone mineral density (BMD) in males and premenopausal women [37]. These reports suggest that lutein is a possible candidate for maintaining bone health; however, the effect of lutein on disuse-induced osteoporosis remains unknown. In addition, the molecular mechanism of lutein for bone cells is not clarified. In this study, we examined the effects of lutein on osteoporotic bone loss in HLU mice. We identified IκB kinase (IKK) β protein as an intracellular target protein of lutein, which blocks IKKβ kinase activity, and lutein showed an ameliorating effect on disuse-induced osteoporosis in HLU mice.

## 2. Materials and Methods

### 2.1. Animals and Reagents

Neonatal, 6-week-old male *ddY* mice and 6-week-old male C57BL/6J mice were obtained from Japan SLC Inc. (Shizuoka, Japan). All procedures were performed in accordance with the institutional guidelines of the Animal Research Committee of the Tokyo University of Agriculture and Technology (protocol number: 28–57). Recombinant human IL-1α was purchased from R&D Systems (Minneapolis, MN, USA). L(+). Ascorbic acid was purchased from Fujifilm Wako Pure Chemical Corp. (Osaka, Japan). β-glycerophosphate disodium salt hydrate was purchased from Merck KGaA (Darmstadt, Germany). Purified lutein (Oriza oil and fat chemical, Aich, Japan and Kemin Industries Inc., Des Moines, IA, USA) was used for the experiments.

### 2.2. Osteoclast Differentiation Induced by IL-1 in Cocultures of Mouse Primary Osteoblasts and Mouse Bone Marrow Cells

Mouse primary osteoblasts (POBs) were collected from the calvarial bones of newborn mice using sequential digestions with enzymes of 0.1% collagenase (Fujifilm Wako Pure Chemical Corp., Osaka, Japan) and 0.2% dispersed (Roche Diagnostics K.K., Tokyo, Japan). Bone marrow cells (BMCs) were isolated by flashing the tibial bone marrow of 6-week-old mice. These two types of cells were co-cultured with or without IL-1 (2 ng/mL) and each carotenoid at 20 μM (lutein: LUT, astaxanthin: AST, and β-cryptoxanthin: CRY) for 7 days, and osteoclasts were stained to detect tartrate-resistant acid phosphatase (TRAP). TRAP-positive multinucleated osteoclasts were counted.

### 2.3. Osteoclast Differentiation Induced by Soluble RANKL in Cultures of Raw264.7 Cells

The osteoclast precursor cell line Raw264.7 was cultured with sRANKL (100 ng/mL) and lutein (3, 10, and 30 μM) for 5 days. Differentiated osteoclasts were stained for TRAP and TRAP-positive multinuclear cells were counted.

### 2.4. TRAP Staining

The TRAP staining solution was prepared by mixing Naphthol AS-Mix phosphate (Merck KGaA, Darmstadt, Germany) and fast red–violet LB salt (Merck KGaA). Cells were fixed with a 10% formalin solution (Fujifilm Wako Pure Chemical Corp.) for more than 10 min. They were then stained with TRAP staining solution for 20 min.

### 2.5. Organ Cultures of Calvariae from Neonatal Mice

Calvariae of neonatal mice were pre-cultured with each carotenoid (20 μM) for 1 day in BGJb medium supplemented with 1 mg/mL of bovine serum albumin (BSA). Then, calvariae were cultured with IL-1 (2 ng/mL) and each carotenoid (20 μM) for another 5 days. The bone-resorbing activity was determined by measuring the calcium concentration in the conditioned medium using the *o*-Cresolphtalein-Complexone (OCPC) method.

### 2.6. Calcified Bone Nodule Formation in POB Cultures

POBs were cultured with β-glycerophosphate (β-GP) and ascorbic acid (AA) in the presence or absence of LUT, CRY, or AST (20 μM, each) for 14 days. Then, the double staining of alizarin red S (ARS) and alkaline phosphatase (ALP) were conducted to detect calcified bone nodules.

### 2.7. mRNA Expression Analysis by Quantitative PCR

Total RNA was collected from the cells. The concentration of total RNA was measured using a NanoDrop Lite (Thermo Fisher Scientific Inc., Waltham, MA, USA). The cDNA was synthesized by reverse transcription from 5 μg of total RNA using a Superscript II pre-amplification system (Thermo Fisher Scientific Inc.). Subsequently, cDNA was amplified using quantitative PCR (qPCR) with primer pairs designed using Primer3Plus (Whitehead Institute for Biomedical Research, Cambridge, MA, USA). The sequences of the mouse PCR primer pairs (synthesized by Eurofins Scientific, Luxembourg) were described in Table 1. qPCR was performed using SsoAdvanced SYBR Green Supermix (Bio-Rad Laboratories Inc., Hercules, CA, USA) using a CFX Connect Real-Time PCR System (Bio-Rad Laboratories Inc.). The relative mRNA expression (ΔΔCq value) was analyzed using Bio-Rad CFX Manager 3.1 (BioRad Laboratories Inc.). All mRNA expressions were normalized by the mRNA expression of β-actin.

### 2.8. In Silico Molecular Docking Simulation

The X-ray crystal structure of the IKKβ protein was referenced from a protein databank (PDB ID:4kik) [38].

A molecular docking study was conducted to elucidate the binding modes and interactions between the synthesized compounds and the target protein. The following protocols were used.

#### 2.8.1. Ligand Preparation

The 2-dimensional chemical structure of lutein was initially constructed using the ChemDraw software program (version 19.0, PerkinElmer Informatics, Waltham, MA, USA). Subsequently, the lutein structure was converted into a 3-dimensional conformation and subjected to energy minimization to obtain its optimal geometries.

#### 2.8.2. Protein Preparation

The crystal structure of the target protein IKKβ (PDB ID: 4kik) was obtained from PDB. The protein structure was processed using MGLtools version 1.5.7 (Center for Computational Structural Biology, La Jolla, CA, USA). Hydrogen atoms were added and the hydrogen bond network was optimized to ensure that the protein structure was suitable for docking simulations.

#### 2.8.3. Binding Site Definition

A 3-dimensional grid box encompassing the key residues of the active site was defined to delineate the search space for the docking simulations.

#### 2.8.4. Molecular Docking

AutoDock Vina (ver. 1.2.5, Center for Computational Structural Biology, San Diego, CA, USA) was used to perform the molecular docking simulations. Docking parameters were meticulously set, including the grid box dimensions, center coordinates, exhaustiveness, and number of output poses.

#### 2.8.5. Post-Docking Analysis

Docking poses were ranked using a scoring function inherent to AutoDock Vina. The highest-scoring conformations were selected for further analyses.

#### 2.8.6. Visualization and Interaction Analysis

The optimal docking poses were imported into Discovery Studio Visualizer (Dassault Systèmes, Vélizy-Villacoublay, France) for detailed examination.

### 2.9. In Vitro Assay of IKK Kinase Activity

The inhibitor of NF-κB kinase (IKK) activity was measured using an IKKα and β Kinase Assay/Inhibitor Screening Kit (Medical and Biological Laboratories Co., Ltd., Nagoya, Japan).

### 2.10. Protein Expression Analysis by Western Blotting

Lysis buffer containing PhosSTOP (Roche) and the complete protease inhibitor cocktail EASYPack (Roche) was treated to the cell culture dish to lyse cells and collect total protein. The protein concentration of the whole cell lysates was measured by a bicinchoninic acid (BCA) protein assay kit (Thermo Fisher Scientific Inc.). Ten micrograms of protein from each sample was loaded in 10% polyacrylamide gel and SDS-PAGE was run. Then, proteins in the gel were transferred onto polyvinylidene difluoride membranes (Merck KGaA) by wet tank transfer. Membranes were cut to appropriate size, blocked with 5% skim milk in 0.05% PBS-T, and incubated with primary antibodies at 4 °C overnight. Membranes were incubated with the secondary antibodies in 1% skim milk in PBS-T and developed with ECL Prime Western Blotting Detection Reagent (GE Healthcare Japan Corp., Tokyo, Japan) using ChemiDoc XRS+ (Bio-Rad Laboratories Inc.). Primary antibodies against phospho-NF-κB p65 (Ser536) (65 kDa; Cell Signaling Technology Inc., Danvers, MA, USA) and β-actin (43 kDa; Santa Cruz Biotechnology Inc., Dallas, TX, USA) were used.

### 2.11. Feeding 1% Lutein-Contained Diets to HLU Mice

Eight-week-old male mice were randomly divided into 4 groups: a control group (n = 5), a 1% lutein-containing diet-fed control group (n = 5), a hindlimb unloading (HLU) group (n = 5), and a 1% lutein-containing diet-fed HLU group (n = 5). In the HLU model, the hindlimbs were always floated in the air, but mice were free to move, eat, and drink using their forelimbs, which were in contact with the floor. Mice were fed a 1% lutein-containing diet. After two weeks, the femoral bone mass was measured by μCT (R_mCT2; Rigaku Corp., Tokyo, Japan). Bone microarchitectural parameters were analyzed using TRI-3D-BON (Ratoc System Engineering Co., Ltd., Tokyo, Japan).

### 2.12. Statistical Analysis

All data are expressed as the median or mean ± standard error (SE) with individual data points. For comparisons among 3 or more groups, a one-way ANOVA followed by Tukey’s test was calculated using GraphPad Prism (ver. 10.3.0 (507), GraphPad Software, San Diego, CA, USA).

## 3. Results

### 3.1. Compared Effects of Lutein, Astaxanthin, and β-Cryptoxanthin on Osteoclastic Bone Resorption

**Figure 1:**
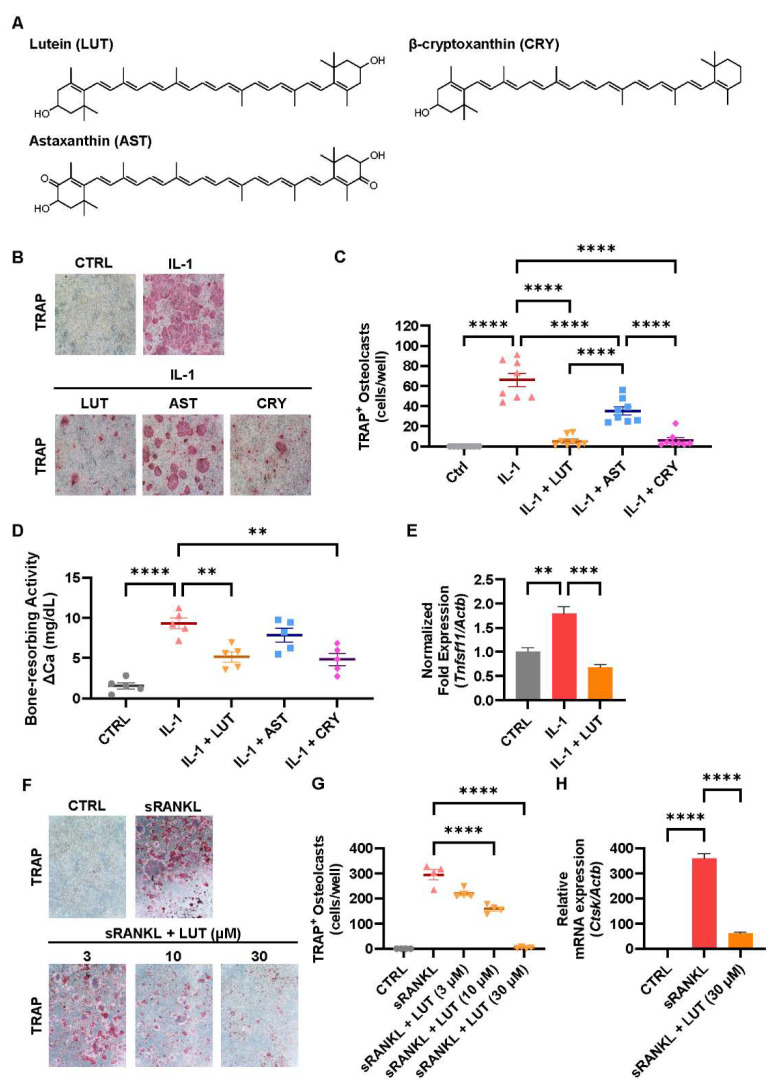
Effects of LUT, CRY and AST on osteoclastic bone resorption. (**A**) Chemical structures of lutein (LUT), β-cryptoxanthin (CRY), and astaxanthin (AST) were described. (**B**,**C**) POBs and BMCs were cocultured with IL-1 (2 ng/mL) and LUT, CRY, or AST (20 μM, each) for 7 days. Images show TRAP-stained multinucleated osteoclasts (**B**). The number of TRAP-stained multinucleated osteoclasts was counted (**C**). (**D**) Calvarial bones from neonatal mice were cultured with IL-1 (2 ng/mL) and each carotenoid (20 μM). Bone-resorbing activity was determined by measuring the concentration of calcium leached from bone in conditioned medium. (**E**) The mRNA expression of *Tnfsf11* (encoding RANKL) was quantified by RT-qPCR. (**F**,**G**) Raw264.7 cells were cultured with or without sRANKL (100 ng/mL) and LUT (3, 10, and 30 μM) for 5 days. Images show TRAP-stained multinucleated osteoclasts (**F**). The number of TRAP-stained multinucleated osteoclasts was counted (**G**). (**H**) The mRNA expression of *Ctsk* (encoding cathepsin K) was analyzed by RT-qPCR. The data are expressed as the mean ± SEM of 8 cultures (**C**), 5 bones (**D**), 4 cultures (**G**), or triplicate from a representative experiment (**E**,**F**). The *Actb* gene was used for normalization. Asterisks indicate significant differences between 2 groups: ** *p* < 0.01, *** *p* < 0.001, **** *p* < 0.0001 by a one-way ANOVA followed by post hoc Tukey’s test.

The chemical structures of lutein, astaxanthin, and β-cryptoxanthin were determined using MarvinSketch (version 24.3.0., Chemaxon Ltd., Advanced Chemical Development, Inc., Budapest, Hungary) (Figure 1A). First, the effects of lutein, astaxanthin, and β-cryptoxanthin on osteoclastic bone resorption were compared. In the cocultures of POBs and BMCs, these 3 carotenoids significantly inhibited IL-1-induced osteoclast differentiation (Figure 1B,C). In mouse calvarial organ cultures, lutein and β-cryptoxanthin significantly suppressed the IL-1-induced bone-resorbing activity, whereas astaxanthin suppressed it (Figure 1D). Lutein significantly downregulated the mRNA expression of *Tnfsf11*, which encodes the RANKL protein in osteoblasts (Figure 1E). In addition, lutein dose-dependently inhibited RANKL-induced osteoclast differentiation associated with the downregulation of the *Ctsk* mRNA expression in Raw264.7 cultures (Figure 1F–H). These results indicated that the inhibitory effects of LUT and CRY were comparable, and both were significantly greater than the inhibitory effects of AST. The inhibitory effect of lutein was mediated through the downregulation of the *Tnfsf11* expression and the direct inhibition of the osteoclast differentiation induced by RANKL.

### 3.2. Effects of Lutein, Astaxanthin, and β-Cryptoxanthin on Osteoblastic Bone Mineralization

**Figure 2:**
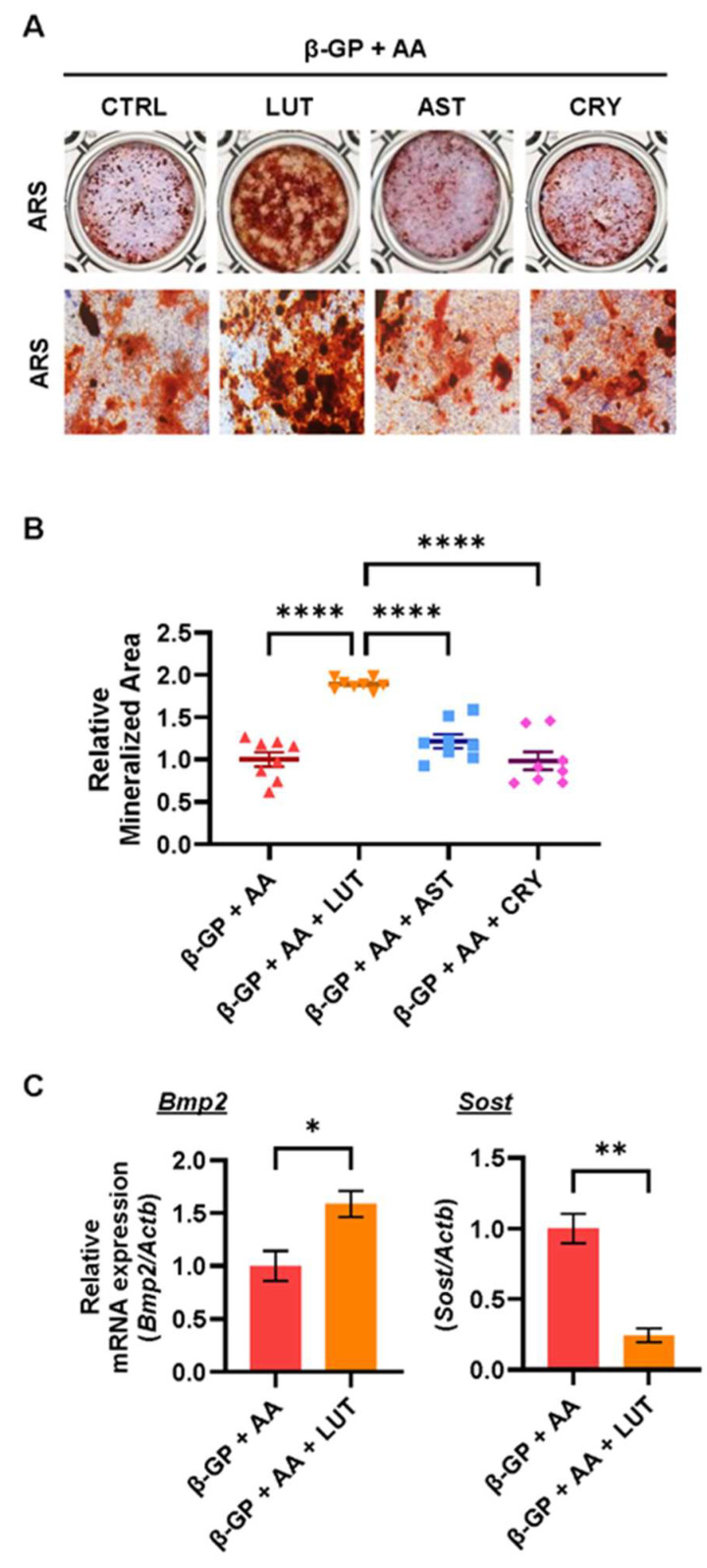
Effects of LUT, CRY and AST on osteoblastic bone formation. (**A**,**B**) POBs were cultured with β-glycerophosphate (β-GP) and ascorbic acid (AA) and LUT, CRY, or AST (20 μM, each) for 14 days. Images of alizarin red S (ARS) and alkaline phosphatase (ALP) double-staining are shown (**A**). The ARS-positive area was measured as the bone mineralized area (**B**). (**C**) The mRNA expression of *Bmp2* and *Sost* (encoding sclerostin) was analyzed by RT-qPCR. The data are expressed as the mean ± SEM of 8 cultures (**B**) or triplicate from a representative experiment (**C**). The *Actb* gene was used for normalization. Asterisks indicate a significant difference between 2 groups: * *p* < 0.05, ** *p* < 0.01, **** *p* < 0.0001 by a one-way ANOVA followed by post hoc Tukey’s test (**B**) and by a two-tailed Welch’s *t* test (**C**).

The effects of lutein, astaxanthin, and β-cryptoxanthin on calcified osteoblastic bone nodule formation in POB cultures were compared. As shown in Figure 2A,B, only lutein significantly promoted calcified bone nodule formation induced by the addition of β-GP and AA, whereas astaxanthin and β-cryptoxanthin did not affect the phenotype. We further examined the mRNA expression of osteogenic genes *Bmp2*, *Sost*, *Osx*, and *Runx2*. Figure 2C shows that lutein significantly upregulated the mRNA expression of *Bmp2*, a cytokine with osteogenic activity, and downregulated that of *Sost*, a glycoprotein with anti-osteogenic activity. These data indicate that lutein plays a unique role in promoting osteoblastic calcified bone nodule formation by regulating the expression of BMP2 and Sclerostin.

### 3.3. Lutein Ameliorated the NF-κB Pathway in Osteoblasts

**Figure 3:**
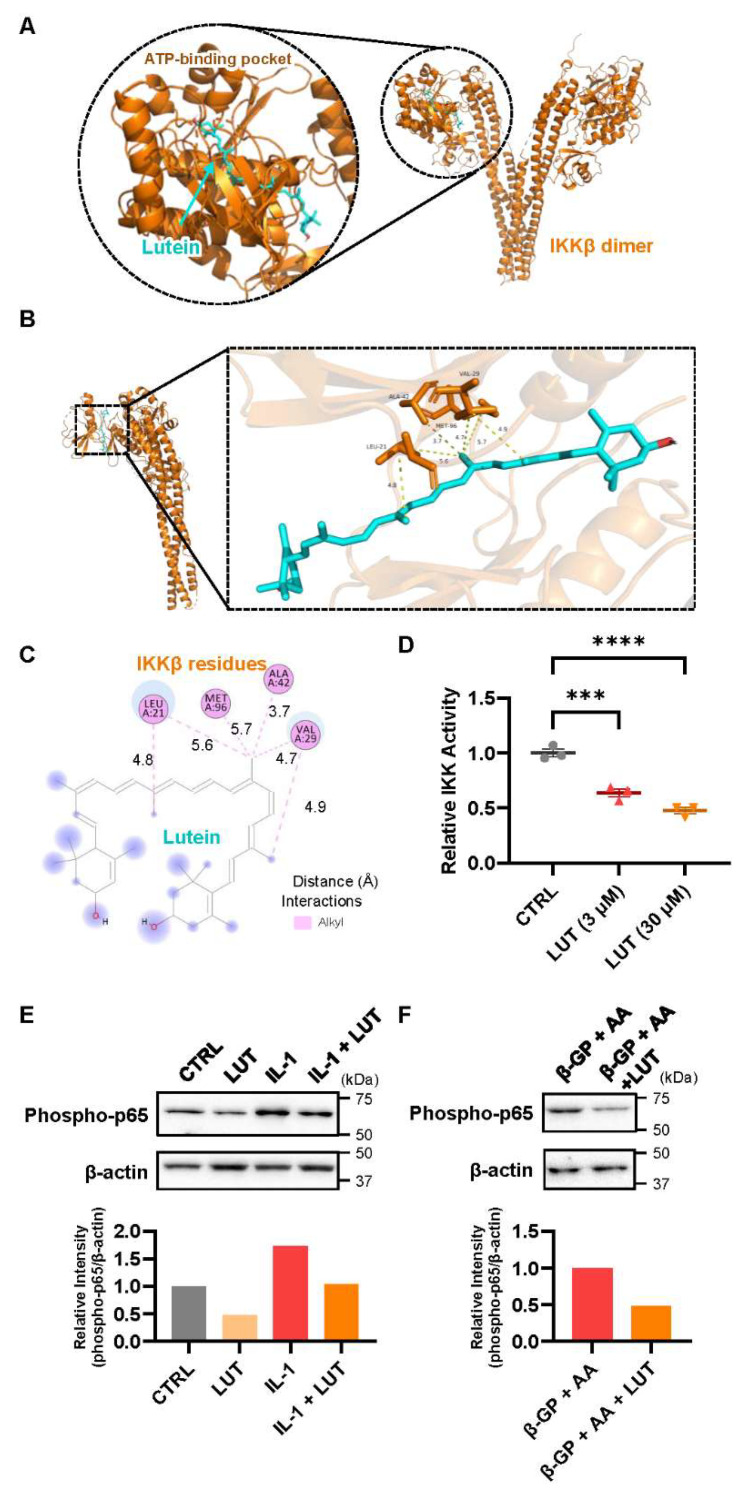
Mechanistic analysis of the effects of lutein on osteoclast differentiation and bone mineralization. (**A**) The overall image of the molecular docking results. (**B**,**C**) Three-dimensional (**B**) and 2-dimensional images (**C**) of the docking site. The dashed lines indicate interactions between hydrophobic regions and charged areas of the molecules. Numbers indicate the distance (Å) between IKKβ residue and lutein. (**D**) The effect of lutein on the kinase activity of IKKβ in an in vitro experiment. The data are expressed as the mean ± SEM of 3 wells. Asterisks indicate a significant difference between 2 groups: *** *p* < 0.001 and **** *p* < 0.0001 by a one-way ANOVA followed by post hoc Tukey’s test. (**E**,**F**) Mouse POBs were treated with IL-1 or β-GP + AA and lutein for 24 h. Whole-cell lysates were collected, and phospho-p65 and β-actin were detected by Western blotting. The blot images are shown in upper images, and the relative blot intensity of phospho-p65 was shown in lower graphs.

To clarify the mechanism underlying the effects of lutein, we examined whether lutein regulates the NF-κB pathway. The IKK complex (IKKα-IKKβ-NEMO) phosphorylates IκBα and the subsequent degradation of IκBα via the ubiquitin-proteasome system, which results in the nuclear translocation and transcriptional activation of NF-κB. We analyzed the possible binding of lutein to the ATP-binding site of the IKKβ protein in an in silico molecular docking test. Figure 3A–C illustrate the binding mode of lutein within the IKKβ protein-binding site. The predominance of hydrophobic interactions with residues Ala42, Leu21, Val29, and Met96 suggests that these amino acids are key determinants of the recognition and binding of lutein (Figure 3B,C). The binding energy of lutein to IKKβ was −7.9 kcal/mol. This binding energy is smaller than −7.0, indicating that these compounds can strongly interact with a target protein. We further examined the direct effect of lutein on the kinase activity of IKKβ in vitro using an enzyme-coated plate and showed that lutein significantly inhibited IKKβ activity (Figure 3D). We further analyzed the protein expression of phosphorylated p65, an active form of p65. As shown in Figure 3E,F, lutein reduced the level of phosphorylated p65 in both cell culture systems. These data indicate that the inhibitory effects of lutein on osteoclast differentiation and the promoting effects on calcified bone nodule formation were partially mediated by the inhibition of the NF-κB pathway.

### 3.4. Feeding of a 1% Lutein-Contained Diet Ameliorated Bone Loss in HLU Mice

**Figure 4:**
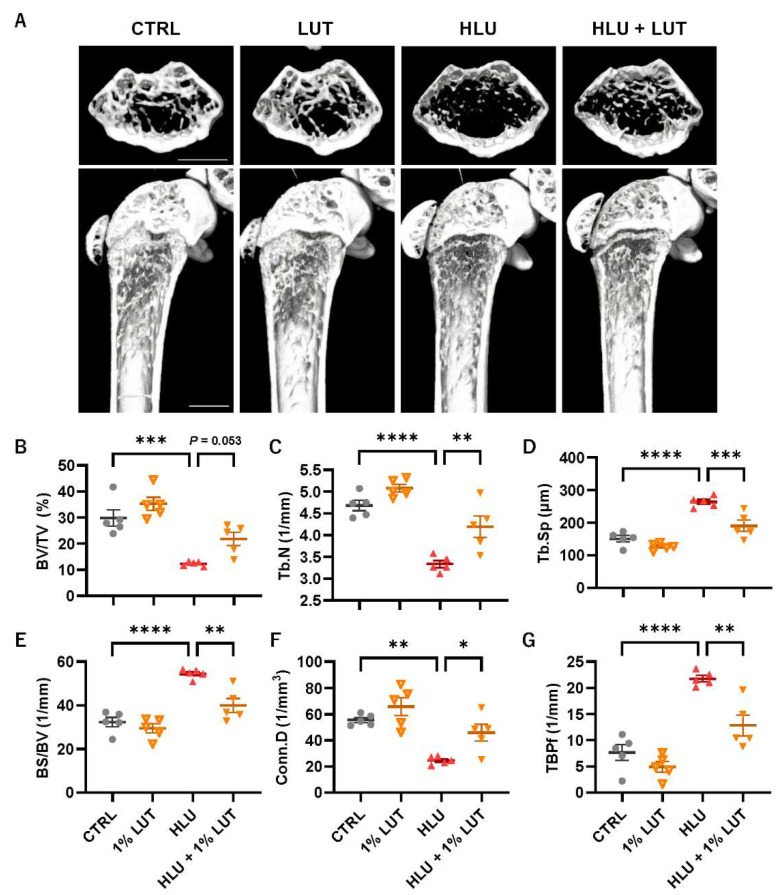
Lutein intake ameliorates disuse-induced bone loss in HLU mice. (**A**) Reconstructed images using μCT of a horizontal section and the distal femur from a longitudinal section. The scale bar represents 1 mm. (**B**–**G**) The bone microarchitecture parameters, BV/TV (%) (**B**), Tb.N (1/mm) (**C**), Tb.Sp (μm) (**D**), BS/BV (1/mm) (**E**), Conn.D (1/mm^3^), and TBPf (1/mm) were calculated by μCT. The data are expressed as the mean ± SEM of 5 mice. Asterisks indicate a significant difference between 2 groups: * *p* < 0.05, ** *p* < 0.01, *** *p* < 0.001, and **** *p* < 0.0001 by a one-way ANOVA followed by post hoc Tukey’s test.

The effect of lutein on disuse-induced bone loss in HLU mice was also examined. Mice were fed a 1% lutein-containing diet for 3 days before HLU and were then exposed to HLU for 14 days. The dietary intake did not change among the groups, and mice were fed lutein at approximately 40 mg/day/mouse in the lutein-feeding groups. A μCT analysis of the hindlimb femoral bone microarchitecture revealed clear bone loss in HLU mice on 3-dimensional μCT images (Figure 4A). This was indicated by a decrease in the bone volume per tissue volume (BV/TV), a reduction in the trabecular number (Tb.N) (Figure 4B,C), an increase in trabecular separation (Tb.Sp), and an increase in bone surface per bone volume (BS/BV) (Figure 4D,E). In addition, HLU exacerbated the bone quality parameters, including the connectivity density (Conn.D) (Figure 4F) and trabecular bone pattern factor (TBPf) (Figure 4G). Lutein feeding significantly improved these parameters (Figure 4), indicating that lutein prevented disuse bone loss in HLU mice.

## 4. Discussion

**Figure 5:**
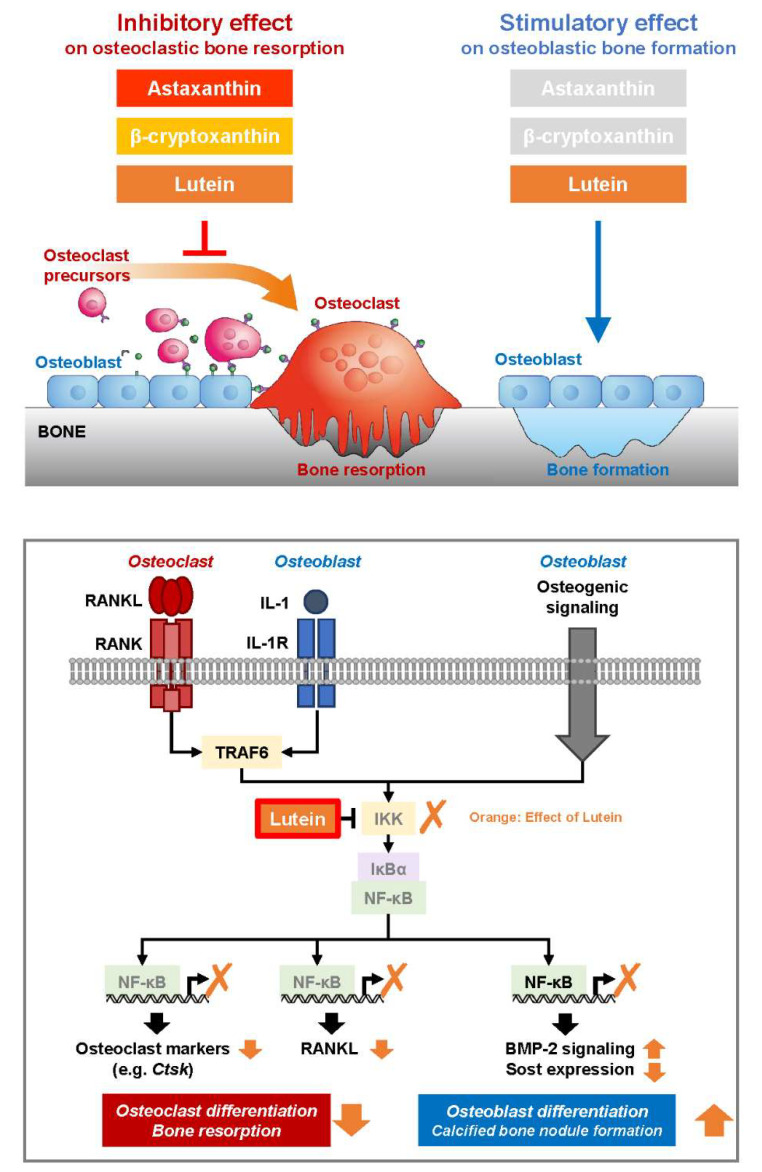
Model illustrating the effects of lutein on bone resorption and bone formation. Three carotenoids, including astaxanthin, β-cryptoxanthin, and lutein, inhibited osteoclast differentiation and bone resorption, whereas only lutein promoted osteoblastic calcified bone nodule formation. Mechanistically, lutein can directly bind to IKK protein and suppress its kinase activity, attenuating NF-κB transcriptional activation. The inhibition of NF-κB by lutein results in the downregulation of osteoclast markers in osteoclasts and RANKL in osteoblasts, which, in turn, inhibits osteoclastic bone resorption. In contrast, the inhibition of NF-κB by lutein can elevate the expression of *Bmp2* and suppress the expression of *Sost*, leading to osteoblastic calcified bone nodule formation.

The present study demonstrated that lutein maintains bone mass in a disuse-induced osteoporosis mouse model of HLU. The effects of lutein, astaxanthin, and β-cryptoxanthin on osteoclast differentiation and osteoblastic calcified bone nodule formation were evaluated in vitro. Molecular mechanisms and the functions of lutein on bone cells are illustrated in Figure 5.

Three carotenoids significantly suppressed osteoclast differentiation in the cocultures of POBs and BMCs (Figure 1B,C) and bone-resorbing activity in mouse calvarial organ cultures (Figure 1D). The inhibitory effect of lutein on osteoclastic bone resorption was mediated by two mechanisms: an indirect mechanism that targeted osteoblasts to suppress the expression of RANKL and a direct mechanism that targeted osteoclast precursor cells (Figure 1E–H). We demonstrated that β-cryptoxanthin downregulated the expression of RANKL mRNA and suppressed RANKL-induced osteoclast differentiation [15,39]. Astaxanthin has been reported to suppress RANKL-induced osteoclast differentiation [40]. In this study, we discovered a suppressive effect of lutein on the expression of RANKL in osteoblasts.

An evaluation of calcified bone nodule formation using the three carotenoids showed that only lutein significantly enhanced calcified bone nodule formation associated with the upregulation of the expression of *Bmp2* and the downregulation of the expression of *Sost* in POB cultures (Figure 2). A previous study reported that β-cryptoxanthin (0.1, 1 μM) promoted osteoblastic cell growth and calcified bone nodule formation in the mouse osteoblastic cell line MC3T3-E1 [41]. Another report indicated that astaxanthin (1.6 and 16 μM) induces the osteogenesis of mesenchymal stem cells [42]. These results raised differences in the cell types or the treated concentrations; however, the effect of lutein on calcified bone nodule formation was most effective.

There are several possible mechanistic targets for the dual effects of lutein on bone tissue. Antioxidative effects are one of these mechanisms. Excessive oxidative stress has been reported to stimulate osteoclast differentiation and function [43,44,45] and decrease osteoblast differentiation and calcified bone nodule formation [43,46,47]. To elucidate the molecular mechanism of lutein, we functionally analyzed NF-κB, a molecule involved in bone metabolism. An in silico molecular docking simulation indicated that lutein is capable of forming hydrophobic-bonding interactions between lutein and residues of IKKβ with Ala42 (3.7 Å), Leu 21 (4.8 Å and 5.6 Å), Val29 (4.7 Å and 4.9 Å), and Met96 (5.7 Å) (Figure 3). To support the docking simulation results, in vitro experiments showed that lutein directly suppressed the kinase activity of IKKβ (Figure 3D). In addition, the level of phosphorylated p65 protein was reduced by lutein (Figure 3E,F). Our previous reports have indicated that β-cryptoxanthin downregulates IL-1- or LPS-induced PGE synthetases, cyclooxygenase-2 (COX-2), and membrane-bound PGE synthase-1 (mPGES-1) in osteoblasts via IKKβ inhibition, thereby inhibiting PGE2-induced RANKL expression and subsequent osteoclast differentiation [15,28,30]. Since NF-κB transcriptionally regulates PGE synthases in osteoblasts and several osteoclast markers, including NFATc1, in osteoclasts, we suggest that the inhibitory effect of lutein on osteoclast differentiation is due to the direct inhibition of the IKK-dependent NF-κB pathway (Figure 5).

In terms of the effects on osteoblastic calcified bone nodule formation, lutein upregulates *Bmp2* expression and downregulates *Sost* expression (Figure 2). Rojasawasthien et al. reported that NF-κB inhibition induced BMP2-induced ectopic bone formation in mice by enhancing BMP signaling [48]. Baek et al. reported that NF-κB directly binds to NF-κB-binding elements on the *Sost* promoter and upregulates *Sost* expression in MLO-Y4 osteocytes [49]. Several studies have indicated that NF-κB inhibitors enhance osteoblast differentiation and calcified bone nodule formation by upregulating osteoblast-specific genes, including *Col1a1*, *Alp*, and *Opn* [50], and by enhancing c-Jun *N*-terminal kinase (JNK) activity [51]. These reports suggest that the inhibition of NF-κB promotes bone formation and lutein promotes calcified bone nodule formation by IKKβ-binding and subsequent NF-κB inhibition.

For other thoughtful explanations for the effects of lutein on osteoclast differentiation and calcified bone nodule formation, lutein exhibits antioxidative properties by directly scavenging ROS, such as singlet oxygen and lipid peroxy radicals [52], and activating the Nrf2 pathway [53,54]. ROS have been reported to be mediators of several osteoclastogenic signaling pathways, such as mitogen-activated protein kinases (MAPKs) and NF-κB in osteoclast precursor cells and osteoclasts [55], and to activate RANKL expression by activating MAPKs, a cAMP response-element-binding protein (CREB), and NF-κB pathways in osteoblasts [56]. Additionally, inhibiting or enhancing ROS scavenging can effectively suppress osteoclast differentiation [44,57]. In contrast, ROS negatively affects osteoblastic bone formation. Excess ROS induce osteoclast apoptosis through the PI3K/AKT/GSK3 and MAPK pathways and RUNX2 destabilization, impairing osteoblast differentiation and calcified bone nodule formation [58,59,60]. Further studies are needed to demonstrate why only lutein showed bone-forming activity rather than other carotenoids, such as astaxanthin and β-cryptoxanthin. The present study suggests that lutein’s attenuation of the NF-κB pathway and oxidative stress contributes to the inhibition of osteoclast differentiation and the promotion of osteoblastic bone formation. Finally, we investigated the effects of lutein on disuse-induced osteoporosis in HLU mice. HLU mice showed femoral bone loss associated with deteriorated bone microarchitecture parameters, but feeding lutein significantly ameliorated these parameters and restored bone loss in HLU mice (Figure 4). To exert significant effects of phytochemicals, such as polymethoxyflavones [16] and their demethylated metabolites [14], on bone loss in mice, repeated intraperitoneal administration was required due to effective absorbance in the mouse body. We previously reported that feeding a 1% lutein-containing diet to growing mice (5-week-old to 9-week-old) increases the femoral bone mass [29]. In this study, the μCT parameters of the bone microarchitecture were evaluated. The trabecular bone volume fraction (BV/TV), trabecular number (Tb.N), and trabecular separation (Tb.Sp) are well-known bone morphometric parameters. The connectivity density (Conn.D) and trabecular bone pattern factor (TBPf) indicate trabecular connectivity, which is a structural property of the trabecular bone that affects bone strength. Feeding a 1% lutein-containing diet improved these parameters in HLU mice with disuse-induced osteoporosis.

## 5. Conclusions

The highlight of this study is that lutein inhibits bone resorption and promotes bone formation in in vitro and in vivo experiments. Lutein improves bone loss due to the disuse osteoporosis model in mice. These outcomes strongly support the application of dietary supplements containing lutein for human diseases. On the other hand, a future challenge is needed to elucidate the molecular biological differences between β-cryptoxanthin and astaxanthin. In particular, β-cryptoxanthin and lutein have also been indicated to bind to IKKβ protein [17]. Therefore, it is suggested that lutein has other target proteins related to signaling pathways on bone formation. A further trial is needed to conduct human studies for lutein, including effective the concentration and absorption rate for human use under the regulatory framework of making functional foods. In conclusion, the present study demonstrates that lutein exhibits an anti-disuse osteoporotic effect in HLU mice. Lutein directly targets the IKKβ protein and blocks its kinase activity, suppressing osteoclast differentiation and promoting bone formation. Lutein is a candidate for promoting bone health and preventing disuse-induced osteoporosis before individuals reach systemic bone fractures.

## Figures and Tables

**Table 1:** Primer sequences for qPCR.

Gene	Forward	Reverse
*Actb*	5′-ccccattgaacatggcattg-3′	5′-acgaccagaggcatacagg-3′
*Tnfsf11*	5′-aggctgggccaagatctcta-3′	5′-gtctgtaggtacgcttcccg-3′
*Ctsk*	5′-cattctcagacacacaatccac-3′	5′-gatactggacaccactggga-3′
*Bmp2*	5′-gtcgaagctctcccactgac-3′	5′-caggaagctttgggaaacag-3′
*Sost*	5′-gtgtgatgttgggctacgtg-3′	5′-ccaccacaatctctccccta-3′

## Data Availability

The original contributions presented in the study are included in the article, further inquiries can be directed to the corresponding author/s.
